# Detection of Posttraumatic Stress Disorder With Rest-Activity Data: Machine Learning Approach Using Wearable and Self-Report Data

**DOI:** 10.2196/86025

**Published:** 2026-05-19

**Authors:** Katherine Wislocki, Ghazal Naderi, Jessica L Borelli, Mark Pollack, Douglas A Granger, David P Cenkner, Margaret Canady, Helen J Burgess, Alyson K Zalta

**Affiliations:** 1 Department of Psychology, University of California, Irvine, 214 Pereira Dr, Irvine, CA, 92617, United States, 1 949 824 5574; 2 Department of Behavioral Sciences and Psychiatry, Rush University Medical Center, Chicago, IL, United States; 3 Department of Pediatrics, Johns Hopkins Medicine, Baltimore, MD, United States; 4 Department of Psychiatry, University of Michigan Medicine, Ann Arbor, MI, United States

**Keywords:** actigraphy, wearables, machine learning, posttraumatic stress, veterans

## Abstract

**Background:**

Growing evidence suggests that disruptions in rest-activity rhythms may serve as relevant markers of posttraumatic stress disorder (PTSD). Despite the emergence of machine learning methods applied to actigraphy and self-report data, few studies have used these approaches to identify individuals with clinically diagnosed PTSD. Prior work has focused on predicting probable PTSD based on self-report measures, yet discrepancies exist between clinical diagnoses and probable PTSD derived from self-reports.

**Objective:**

This study explored whether wrist actigraphy and sleep logs could be used to accurately predict clinician-rated PTSD diagnosis and probable diagnosis of PTSD based on established self-report cutoffs (PTSD Checklist for *Diagnostic and Statistical Manual of Mental Disorders, Fifth Edition* [PCL-5] ≥31 and ≥38) among trauma-exposed service members and veterans. We also explored which features were most strongly predictive of each outcome and whether models were able to predict PTSD diagnosis even when accounting for other mental health disorders.

**Methods:**

Wrist actigraphy data and daily sleep logs were collected over 1 week from trauma-exposed male service members and veterans (N=36; mean age 41, SD 5.3 y). Candidate features were identified using univariate feature selection. Extreme gradient boosting models were trained using leave-one-subject-out cross-validation to predict the diagnosis of PTSD and probable diagnosis of PTSD based on 2 self-report cutoffs (PCL-5≥31 and ≥38). Performance metrics were then calculated at the person level. Linear regression was used to assess the discriminant validity of model-predicted scores and each PTSD outcome specifically, relative to other mental health diagnoses.

**Results:**

Machine learning models predicting PTSD diagnosis and probable PTSD based on the PCL-5≥31 threshold demonstrated satisfactory performance in this sample. The diagnosis model achieved an area under the curve (AUC) of 0.83 (95% CI 0.61‐1.00), with high accuracy (88%) and specificity (96%) and moderate sensitivity (63%). The PCL-5≥31 model yielded comparable performance (AUC=0.84, 95% CI 0.71‐0.98) with balanced sensitivity (73%) and specificity (82%). For both models, a combination of subjective and objective features was the most impactful. These models were able to predict PTSD even when accounting for non-PTSD mental health diagnoses, as model-predicted scores were significantly associated with 2 outcomes: clinician-rated PTSD (B=0.19; *P*=.002) and probable PTSD based on a PCL-5≥31 cutoff (B=0.24; *P*=.003). In contrast, the model predicting probable PTSD based on the PCL-5≥38 threshold performed poorly (AUC=0.47, 95% CI 0.24‐0.69), with a nonsignificant relationship between model-predicted scores and the outcome (B<0.01; *P*=.89).

**Conclusions:**

Both subjective and objective rest-activity features may improve the prediction of PTSD. Further research is needed to validate these findings and explore the use of integrating wearable sensor data and subjective information to support PTSD assessment.

## Introduction

The circadian system is the intricate internal clock that aligns various biological processes in accordance with the 24-hour cycle of day and night [1]. Disruptions in one’s circadian rhythm can impact the functioning of different physiological processes, including those related to sleep regulation, metabolic functioning, and hormone secretion [1]. Increasing research has demonstrated evidence of circadian disruption, or “disturbances in biological timing,” in individuals experiencing mental health issues, including depression [2], bipolar disorder [3], schizophrenia [4], and posttraumatic stress disorder (PTSD) [5,6]. Past work has indicated that circadian disruption may be central to the development and maintenance of PTSD [1,7-9]. Trauma exposure, and the onset of traumatic stress, may result in short- and long-term circadian disruption [1,8]. In turn, circadian disruption is thought to potentially mediate the neurobiological consequences associated with traumatic stress [1,8,9].

Research has increasingly focused on using objective forms of measurement to capture information regarding circadian disruption through individual rest-activity rhythms [1,10]. This has become more feasible with the introduction of technology capable of passively sensing objective data related to rest and activity [11]. Actigraphy is a promising tool for passively sensing information regarding rest and activity patterns [11], and research has demonstrated that actigraphy can reliably capture certain objective information about rest and activity [12]. In addition, wrist actigraphy provides greater objectivity in capturing sleep characteristics than self-report [13]. For example, a prior study comparing the use of self-reported sleep characteristics (eg, sleep onset, sleep offset, and nightly awakenings) and actigraphy to capture nightly sleep information in a veteran sample demonstrated that less than half of participants demonstrated adequate reliability between self-report of sleep characteristics and sleep characteristics captured through actigraphy [13]. By providing continuous, real-time data on physical activity and sleep, actigraphy offers an approach to feasibly understanding information about rest-activity rhythms in individuals with PTSD.

Historically, actigraphy data have been used to understand sleep-related impairments in individuals with PTSD [11,14, 15, 16, 17]. Numerous studies have found little difference in sleep-related actigraphy measures between individuals with and without PTSD, including total sleep time [17], sleep onset latency [16,17], nocturnal awakenings [16,17], and sleep efficiency [15,17]. In contrast, some research has demonstrated specific differences in rest-activity rhythms in individuals with PTSD compared to other clinical populations and healthy controls [5,18], such as significantly greater frequency of awakenings during sleep and variability in daily activity levels [18]. Beyond specific rest-activity metrics, the timing of rest and activity patterns is also particularly important for identifying and understanding rest-activity rhythms among individuals with PTSD symptoms. For example, Sandahl et al [6] previously found a significant positive association between PTSD symptoms and the timing of the onset of the most active period of the day, such that individuals with more severe PTSD symptoms were more likely to be most active later in the day compared to earlier in the day. In contrast to healthy controls, individuals with PTSD have also been shown to demonstrate lower relative amplitude (ie, the ratio between daytime and nighttime activity levels) and day-to-day stability in activity [5]. A recent meta-analysis of 17 studies encompassing 1847 participants demonstrated that relative amplitude was significantly related to PTSD symptoms [19]. As such, differences in rest-activity rhythms, as well as the variability in those rhythms, may be helpful in identifying individuals with PTSD.

Actigraphy data can provide access to thousands of measurements per day related to rest, sleep, and light exposure [10,20]. Common measures related to rest-activity rhythms can be nonlinear (eg, acrophase) or linear (eg, mean activity during sleep). Analytic methods must be able to effectively accommodate the breadth, scope, and nature of actigraphy data. Machine learning methods have been used to analyze actigraphy data [21-23]. Past work has used health data (eg, self-report and medical record data) and actigraphy data to help identify individuals with mental health conditions other than PTSD, such as depression, through machine learning [22]. However, limited previous research has used actigraphy data and machine learning to successfully predict PTSD-related outcomes [21,24]. Accurately identifying individuals with a PTSD diagnosis is critical for efforts to assess and intervene to address their symptoms. Rest-activity data may provide insights into the assessment of PTSD symptoms beyond traditional assessment methods, providing an opportunity to differentiate individuals with and without PTSD with reduced burden.

One past study used wrist actigraphy data to predict probable PTSD (ie, defined as PTSD Checklist for *Diagnostic and Statistical Manual of Mental Disorders, Fifth Edition* [PCL-5] scores≥31) in a national sample 2 months following exposure to traumatic events, resulting in a mean accuracy rate of 56% and a mean area under the curve (AUC) value of 0.56 [21]. Rest-activity features, including intradaily variability of activity periods, interdaily stability in rest periods, interdaily stability in activity periods, and circadian rhythm strength, were among the most important actigraphy features in predicting different PTSD outcomes at 8 weeks [21]. When combining both baseline self-report surveys focused on stress and trauma, and wrist actigraphy data, performance in predicting probable PTSD increased (AUC=0.73) [21]. However, the nationwide sample from this study obfuscates how rest-activity data can be used to assess PTSD among other populations, including those at greater risk of experiencing trauma exposure and developing PTSD, such as veterans [25]. More research is needed to understand whether the use of actigraphy data can effectively differentiate individuals with and without PTSD, particularly in individuals with clinically diagnosed PTSD in populations at higher risk for PTSD.

Notably, past research has used a common threshold for establishing a probable PTSD diagnosis through self-report (ie, PCL-5≥31). Research has demonstrated that self-report assessments of PTSD may not always align with clinical assessments of PTSD [26], and self-reported PTSD symptoms are highly variable across time [27,28]. Furthermore, existing research has established a range of “optimal” diagnostic cutoff scores depending on the sample and context of the validation study (eg, PCL-5 scores ranging from 22‐49) [29]. As a result, predicting a probable diagnosis based on a self-report cutoff may not fully reflect the effectiveness of actigraphy in identifying individuals who may meet formal diagnostic criteria for PTSD. Moreover, probable diagnoses based on self-report cutoffs and predicting clinical diagnoses based on clinician-administered interviews represent distinct outcomes; for example, self-report scores offer greater insights into symptom severity, while clinician-administered interviews represent formal clinical evaluations of symptoms based on existing diagnostic standards.

This study is a secondary analysis of wrist actigraphy data and sleep logs in trauma-exposed service members and veterans. Information from wrist actigraphy data and sleep logs from trauma-exposed service members and veterans were used to predict PTSD diagnosis through diagnostic interviews and probable PTSD diagnosis through self-report (PCL-5≥31 and ≥38). While past research has demonstrated a range of potential self-report cutoff scores for identifying PTSD based on self-report (eg, 22‐49), PCL-5 cutoffs of 31 and 38 have been identified as potential diagnostic cutoffs in military populations [29]. We hypothesized that the prediction of PTSD diagnosis and probable PTSD diagnosis would be fair (AUC≥0.70). Feature selection was conducted to assess the most important features for predicting PTSD diagnosis through diagnostic interviews and probable PTSD diagnosis through self-report. To determine whether models could uniquely predict PTSD rather than mental health symptoms more broadly, the associations between co-occurring mental health diagnoses and model predictions were assessed to understand the discriminant validity of wrist actigraphy and sleep logs in differentiating those with PTSD and other mental health concerns. It was hypothesized that there would be adequate discriminant validity, demonstrated through stronger relationships between PTSD diagnosis and probable PTSD diagnosis based on self-report and model-predicted scores, compared to the relationship between non-PTSD mental health diagnosis and model-predicted scores for each model.

## Methods

### Procedures

This study was a secondary analysis of data from a study focused on the intergenerational transmission of trauma among service members, veterans, and their families. Military families (N=36; ie, composed of a male-identifying veteran, one of the veteran’s biological children, and the child’s biological mother) were recruited through several methods, including digital recruitment via social media; ResearchMatch; listservs; and in-person recruitment through schools, local communities, and resource fairs. Participants were eligible to participate if they were male veterans or service members deployed in service of US military, had previously been exposed to a *Diagnostic and Statistical Manual of Mental Disorders, Fifth Edition* (DSM-5) criterion A traumatic event during military service, had a biological child aged 7 to 16 years, were at least aged 18 years, and were fluent in English. Though the initial inclusion criteria required participants to have an index trauma within the context of their military service (determined via phone screening), several participants identified a different index trauma during their clinical interview. These participants were retained in the sample given that all participants had a history of military-related criterion A trauma. Participants were excluded if they had a lifetime history of mania or psychosis, significant cognitive impairment, significant suicidality (defined as suicidal ideation or behavior in the past 6 months), or significant unstable illness. The analysis sample included 33 participants, who had a mean age of 41.36 (SD 5.28) years and an average PCL-5 score of 22.88 (SD 19.04). A total of 8 (24.24%) participants met the criteria for PTSD diagnosis, while 11 (33.33%) and 7 (21.21%) participants met or exceeded the PCL-5 cutoffs of 31 and 38, respectively. Additional information regarding the analysis sample can be found in Table 1.

**Table 1.** Demographic characteristics of participants (n=33)^a^.

Variable	Participants, n (%)	
Race		
Asian	2 (6.06)	
Black or African American	2 (6.06)	
Mixed race or other	4 (12.12)	
White	25 (75.76)	
Ethnicity		
Hispanic, Latino, or Spanish origin	7 (21.21)	
Employment status		
Employed full time	27 (81.81)	
Retired	1 (3.03)	
Student and employed	2 (6.06)	
Full-time student	3 (9.09)	
Highest degree earned		
High school diploma or GED^b^	5 (15.15)	
Some college or associate’s degree	5 (15.15)	
Baccalaureate degree	14 (42.42)	
Master’s degree	9 (27.27)	
Annual income (US $)		
0-49,999	2 (6.06)	
50,000-99,999	11 (33.33)	
100,000-149,999	11 (33.33)	
≥150,000	9 (27.27)	
Index trauma on SCID-5^c^		
Combat	29 (87.87)	
Accidental or violent death	3 (9.09)	
Physical assault	1 (3.03)	
Non-PTSD^d^ mental health diagnosis	9 (27.27)	
Current mental health diagnoses		
PTSD	8 (24.24)	
Major depressive disorder	4 (12.12)	
Generalized anxiety disorder	4 (12.12)	
Social anxiety disorder	4 (12.12)	
Alcohol use disorder	3 (9.09)	
Specific phobia	2 (6.06)	
Persistent depressive disorder	1 (3.03)	
Obsessive-compulsive disorder	1 (3.03)	
Agoraphobia	1 (3.03)	
PCL-5^e^		
≥31	11 (33.33)	
≥38	7 (21.21)	

a Index trauma information was not available through the SCID-5–Research Version assessment for 1 participant as they were unable to complete it. As such, index trauma for this participant was established through the PCL-5.

b GED: General Education Development.

c SCID-5: Structured Clinical Interview for *Diagnostic and Statistical Manual of Mental Disorders, Fifth Edition*.

d PTSD: posttraumatic stress disorder.

e PCL-5: Posttraumatic Stress Disorder Checklist for *Diagnostic and Statistical Manual of Mental Disorders, Fifth Edition*.

Participants were initially scheduled for a 1-hour virtual onboarding session and were shipped a research kit containing an actigraphy watch. During the onboarding session, participants received instructions on how to wear the actigraphy watches and complete daily sleep logs during the 1-week study period. At the end of the onboarding session, participants were emailed self-report assessments for them to complete. Throughout the week, participants received reminders from study staff to complete the self-report assessments. Participants were also scheduled for a diagnostic interview consisting of the Structured Clinical Interview for DSM-5 Disorders–Research Version (SCID-5-RV) [30] with a trained graduate student assessor during the 1-week study period. At the end of the 1-week period, participants mailed the actigraphy watches back to the study team. The actigraphy data were processed by the study team using the Philips Actiware 6.1.0 program (Philips Respironics).

Wrist actigraphy data and sleep logs from participants were used to derive daily features related to individual sleep (eg, sleep efficiency, wake after sleep onset [WASO], subjective sleep quality, and subjective restfulness), activity (eg, mean and SD of activity), and rest-activity patterns (eg, acrophase time and intradaily stability). Given the small sample size, candidate features were identified through univariate feature selection using aggregated features from the full sample. In turn, extreme gradient boosting (XGBoost) models used daily features to try to differentiate individuals with and without diagnosed PTSD and probable PTSD based on self-report cutoffs. Model performance was then evaluated at the person-level across models. The most important predictive features for each model were identified using mean Shapley Additive Explanations (SHAP) values and mean information gain values. To determine the discriminant validity of the model in predicting PTSD diagnosis rather than mental health problems more generally, linear regression analysis was used to assess the relationship between PTSD diagnosis (ie, based on clinical diagnosis or probable diagnosis via self-report) and model-predicted scores while accounting for other non-PTSD mental health diagnoses and other relevant covariates (ie, age).

### Ethical Considerations

Informed consent was obtained from all participants during their onboarding session. Participants were compensated up to US $220 for their participation in this study. Procedures for this study were approved by the institutional review board at the University of California, Irvine (20184726). Additional approval was not required for secondary data analysis. Participant identifiers were stored on encrypted servers, separate from deidentified research data.

### Measures

#### PTSD Checklist for DSM-5

The PCL-5 assesses the severity of PTSD symptoms in the past month based on an individual’s self-reported index trauma [31]. Although all participants experienced trauma within the context of their military service, participants were permitted to identify an index trauma related or unrelated to their military service. The PCL-5 was used to evaluate an individual’s PTSD symptom severity in the past month at baseline. PCL-5 scores greater than or equal to 31 and 38 were evaluated as probable PTSD diagnosis cutoff. Past research has identified various optimal PCL-5 cutoff scores for identifying individuals who may meet the criteria for PTSD, including PCL-5≥31 and PCL-5≥38 in military populations [29].

#### Structured Clinical Interview for DSM-5 Disorders–Research Version

The SCID-5-RV was conducted by trained graduate-level clinical assessors to assess lifetime and current mental health diagnoses [30]. All graduate students conducting assessments received training from the lead author, a clinical psychologist. The assessors conducted a mock screening that was reviewed by the lead author and rated for fidelity prior to conducting study assessments. The lead author also watched and rated recordings of the first study assessment for all assessors to ensure fidelity. Subsequently, the assessors received regular supervision to ensure consistency across raters. Although uncommon, participants were permitted to identify an index trauma through the SCID-5-RV assessment that differed from the index trauma reported through the PCL-5. SCID-5-RV ratings were used to establish a current PTSD diagnosis. These ratings were also used to establish any current diagnosis other than PTSD, including major depressive disorder, persistent depressive disorder, hypomanic episode, manic episode, cyclothymic disorder, generalized anxiety disorder, panic disorder, agoraphobia, social anxiety disorder, specific phobia, obsessive-compulsive disorder, and substance use disorders (coded as 1 for any current non-PTSD diagnosis or 0 for no current non-PTSD diagnosis). Additionally, the SCID-5-RV was used to identify and exclude individuals with a lifetime history of psychosis-spectrum symptoms.

#### Wrist Actigraphy

Wrist actigraphy data were captured for a period of seven days through wrist actigraphy watches (Actiwatch Spectrum Plus, Philips Respironics) worn by participants. Wrist actigraphy watches measured activity and sleep data in 30-second intervals across the study period. Epoch-level data for each participant were exported from the Actiware program. The following features were derived from wrist actigraphy data: mesor, acrophase time, amplitude, relative amplitude, mean activity, SD of activity, root mean square successive difference of activity, intradaily variability of activity, interdaily stability of activity, mean activity during the 10 most active hours of the day (M10), mean activity during the 5 least active hours of the day (L5), circadian rhythm strength, total sleep time, sleep efficiency, WASO, and fragmentation. Total sleep time, sleep efficiency, WASO, and fragmentation were calculated through the Philips Actiware 6.1.0 program, while relative amplitude, mean activity, SD of activity, root mean square successive difference of activity, intradaily variability of activity, interdaily stability of activity, mean activity during the 10 most active hours of the day (M10), mean activity during the 5 least active hours of the day (L5), and circadian rhythm strength were calculated through R version 4.3.1 (R Foundation for Statistical Computing) [32]. Mesor, acrophase time, and amplitude were calculated using the *CosinorPy* package in Python (Python Software Foundation) [33]. Definitions of each feature can be found in Table S1 in Multimedia Appendix 1.

#### Sleep Logs

Participants rated their subjective sleep quality and restfulness each day during the study using a 1-10 scale (1=“worst” to 10=“best”). Sleep logs were completed each morning after waking.

### Analysis

All preprocessing and analysis were conducted in Python [34] and R statistical software [32]. Wrist actigraphy data were used to compute daily features for each individual. Features derived from cosinor-based rhythmometry and the engineering of rest-activity data were used as predictors in each model (see “Measures” section). One participant was excluded from all analyses due to night shift work (n=1), 2 participants were excluded based on a lifetime history of psychosis-spectrum symptoms (n=2), and 1 participant was excluded from diagnostic-specific analysis as they did not complete a SCID-5-RV assessment (n=1). Two participants completed the study during a time change (ie, daylight savings time; n=2). As such, days within 2 days after the initial time change were excluded [35]. Actigraphy data from days with increased off-wrist time (ie, ≥30% off-wrist time) were excluded from analyses. Days with missing sleep log data (n=1) were excluded from analyses. Furthermore, days in which circadian rhythm measures (eg, acrophase time, mesor, and amplitude) were calculated over a period of less than 24 hours were excluded to ensure the reliability of feature estimation. Because of this, the first study day (day 1) and the last study day (day 7) were removed, resulting in 5 days of available data per person. This resulted in approximately 165 days of data for each model. Correlation analysis between variables is presented in Table 2.

**Table 2.** Correlations between rest-activity features and posttraumatic stress disorder (PTSD) outcomes^a^.

Variables	PTSD diagnosis	PCL-5^b^ total	PCL-5≥ 31	PCL-5≥ 38	Non-PTSD diagnosis
PTSD diagnosis	—				
PCL-5 total	.50^c^	—			
PCL-5≥31	.39^d^	.87^e^	—		
PCL-5≥38	.46^c^	.81^e^	.73^e^	—	
Non-PTSD diagnosis	.28	.24	.14	.18	—
Mesor	.04	.15	.18	–.02	.18
Acrophase time	.33	–.04	–.23	–.08	.13
Amplitude	–.01	.05	.08	–.12	.10
Relative amplitude	.07	–.13	–.16	–.22	–.27
Mean activity	.07	.20	.26	.02	–.01
SD activity	.04	.10	.17	–.02	–.05
CRS^f^	.12	–.06	–.06	–.16	–.14
M10^g^	.10	.18	.19	–.01	.04
L5^h^	–.20	.07	.13	.06	.22
RMSSD^i^	.03	.07	.18	–.02	–.02
TST^j^	.14	–.18	–.19	–.12	–.22
IV^k^	.06	.17	.11	.34	.14
IS^l^	–.18	–.18	–.03	–.32	–.10
Sleep quality rating	–.38^d^	–.29	–.33	–.21	–.05
Restfulness rating	–.43^d^	–.44^d^	–.46^c^	–.40^d^	–.11
WASO^m^	.26	.13	.15	.10	.06
Efficiency	–.04	–.36^d^	–.35^d^	–.13	–.31
Fragmentation	.21	.35^d^	.28	.23	.34

a Correlations were calculated among averaged daily features and mental health variables.

b PCL-5: Posttraumatic Stress Disorder Checklist for *Diagnostic and Statistical Manual of Mental Disorders, Fifth Edition*.

c *P*<.01.

d *P*<.05.

e *P*<.001.

f CRS: circadian rhythm strength.

g M10: mean activity during the 10 most active hours of the day.

h L5: mean activity during the 5 least active hours of the day.

i RMSSD: root mean square of successive differences.

j TST: total sleep time.

k IV: intradaily variability.

l IS: interdaily stability.

m WASO: wake after sleep onset.

Due to the limited sample size, univariate feature selection using the *F*-score was conducted on aggregated features for the full sample. Features with higher average *F*-scores were considered to have stronger discriminatory power in predicting each outcome. Given the small sample size, the top 3 features identified through this procedure, with no elevated correlation with another selected feature, were then used across all XGBoost model training folds for each outcome. (ie, *r*≥0.70; Table S2 in Multimedia Appendix 2). For example, this led to the exclusion of the subjective sleep quality rating when the restfulness rating was included, as these features were highly correlated with each other (*r*=0.91). Univariate relationships between each outcome and aggregated features across the full sample are presented in Multimedia Appendix 3. Given the small sample size, a total of 3 features were selected per model to maximize stability in model performance and mitigate model overfitting (Table S3 in Multimedia Appendix 3).

XGBoost models used selected day-level features from wrist actigraphy data and sleep logs to predict PTSD diagnosis and probable PTSD diagnosis via validated PCL-5 cutoffs (ie, PCL-5≥31 and PCL-5≥38). XGBoost is a machine learning algorithm that uses an ensemble learning method by leveraging the performance of individual decision trees to make predictions [36]. In comparison to deep learning models, XGBoost is often more parsimonious and computationally efficient while providing robust and accurate predictions in a variety of contexts, including high-dimensional data and data containing nonlinear relationships [36]. Prior work has used XGBoost to predict mental and physical health outcomes based on passively sensed data [37,38]. Furthermore, past research by Choi et al [22] demonstrated that in comparison to other machine learning and statistical models tested, XGBoost provided the most accurate performance in predicting depression diagnosis based on actigraphy data.

Training and testing data were transformed using a Yeo-Johnson transformation prior to being used to train and test each XGBoost model. Hyperparameters were optimized using randomized grid search. Information about hyperparameters for each model can be found in Table S4 in Multimedia Appendix 4. XGBoost models were trained using leave-one-subject-out cross-validation. In each fold, 1 participant’s day-level data served as the test set, while day-level data from the remaining participants was used for model training. This process was repeated until each participant’s data was used in testing, ensuring that no participant’s data appeared in both training and testing within any fold. Model-predicted scores were then averaged within-person for each XGBoost model and performance statistics for each model were calculated, including area under the receiver operating characteristic curve (accuracy, sensitivity, specificity, precision, and *F*_1_-score). Mean SHAP and information gain values were computed and presented to evaluate the importance of each feature in predicting the outcome. It was hypothesized that the prediction of PTSD diagnosis and probable PTSD diagnosis based on wrist actigraphy and sleep log variables would be fair in this study (AUC≥0.70). SHAP values correspond to the marginal importance of each feature, in context with all other features, in establishing each model prediction [39]. Information gain values reflect the value of each feature in reducing model entropy. Bootstrap tests (using 2000 iterations) were used to compare the AUC values for each model.

Discriminative performance of model predictions in specifically predicting diagnosed and probable PTSD, rather than other mental health outcomes, was assessed using linear regression. For each outcome, a linear regression was conducted using each PTSD-specific outcome (ie, diagnosed PTSD and probable PTSD) to predict XGBoost model-predicted probability scores per person, while covarying for other non-PTSD mental health diagnoses (1=“present” or 0=“absent”). It was hypothesized that there would be adequate discriminant validity, demonstrated through stronger relationships between PTSD diagnosis and probable PTSD diagnosis based on self-report and mean model-predicted probability scores, compared to the relationship between non-PTSD mental health diagnoses and mean model-predicted probability scores for each model. As past work has demonstrated a significant association between PTSD risk and age among male-identifying veterans [40], age was additionally included as a covariate in each linear regression model. It was hypothesized that there would be adequate discriminant validity, demonstrated through stronger relationships between PTSD diagnosis and probable PTSD diagnosis based on self-report and model-predicted scores, compared to the relationship between non-PTSD mental health diagnoses and model-predicted scores for each model.

Logistic regression was additionally used to further evaluate the relationships between the identified features and each outcome. The identified features were averaged within-person and then entered into a logistic regression model for each outcome. Model fit was evaluated using the Nagelkerke *R*².

## Results

### Overview

Correlations between features and outcomes can be found in Table 2. Feature selection results are presented in Table S3 in Multimedia Appendix 3. PTSD diagnosis was strongly correlated with total PCL-5 scores (*r*=0.50; *P*=.003). There was a marginally significant association between current PTSD diagnosis and the PCL-5≥31 cutoff (χ²_1_=3.10; *P*=.08). There was a significant relationship between current PTSD diagnosis and the PCL-5≥38 cutoff (*χ*²_1_=4.38; *P*=.04). Logistic regression results are summarized in Table S5 in Multimedia Appendix 5.

### Diagnosed PTSD

Three features were selected for the PTSD diagnosis model: restfulness rating, WASO, and acrophase time (Table S3 in Multimedia Appendix 3). The model performance metrics are presented in Table 3.

The diagnostic model achieved a satisfactory AUC of 0.83 (95% CI 0.61‐1.00), an accuracy of 88%, a sensitivity of 63% (5/8), a specificity of 96% (24/25), a precision of 83%, and an *F*_1_-score of 0.71. As performance metrics were calculated per person, the precision of these metrics (ie, demonstrated by broad CIs) was impacted by the smaller sample size. Restfulness rating was the most influential predictor (mean SHAP=0.90; information gain=0.39), followed by WASO (SHAP=0.46; information gain=0.32) and acrophase time (SHAP=0.49; information gain=0.30).

**Table 3.** Extreme gradient boosting (XGBoost) model results.

Metric or feature	PTSD Diagnosis Model	PCL-5^a^≥31 Model	PCL-5≥38 Model
AUC^b^ (95% CI)	0.83 (0.61‐1.00)	0.84 (0.71‐0.98)	0.47 (0.24‐0.69)
Accuracy	0.88	0.79	0.61
Sensitivity^c^	0.63	0.73	0.43
Specificity^d^	0.96	0.82	0.65
*F*_1_-score^e^	0.71	0.70	0.32
Precision^f^	0.83	0.67	0.25
Mean SHAP^g^^h^			
Feature 1	Rest Rating=0.90	Efficiency=1.38	Rest Rating=0.42
Feature 2	Acrophase=0.49	Fragmentation=1.35	Fragmentation=0.26
Feature 3	WASO^i^=0.46	Rest Rating=0.92	IV^j^=0.20
Mean information gain^k^			
Feature 1	Rest Rating=0.39	Efficiency=0.48	Rest Rating=0.60
Feature 2	WASO=0.32	Fragmentation=0.38	IV=0.20
Feature 3	Acrophase=0.30	Rest Rating=0.14	Fragmentation=0.20

a PCL-5: Posttraumatic Stress Disorder Checklist for *Diagnostic and Statistical Manual of Mental Disorders, Fifth Edition*.

b AUC: area under the curve.

c Sensitivity is the proportion of true positives correctly identified.

d Specificity is the proportion of true negatives correctly identified.

e *F*_1_-score is the harmonic mean of precision and recall.

f Precision is the proportion of predicted positives that are true positives.

g SHAP: Shapley Additive Explanations.

h Mean SHAP quantifies the average marginal contribution of each feature to individual predictions. Mean SHAP for each feature is organized in rank from largest to smallest.

i WASO: wake after sleep onset.

j IV: intradaily variability.

k Mean information gain reflects how much a feature reduces model entropy when used to split the data during training. Mean information gain for each feature is organized in rank from largest to smallest.

Linear regression analysis revealed a significant positive association between model-predicted scores and clinician-rated PTSD diagnosis (B=0.19, *P*.002), such that individuals with PTSD had predicted probability scores 0.19 points higher on average than those without (Table 4).

However, the model explained only 21% of the variance in predicted scores (adjusted *R²*=0.21). Age and current non-PTSD mental health diagnosis were not significant predictors (*P* values>.05; Table 4).

**Table 4.** Relationship between model-predicted score and posttraumatic stress disorder (PTSD) outcomes.

Variable^a^	B^b^	SE	*t*	*P value*	*R*²^c^
PTSD diagnosis Model					0.21^d^
Intercept	0.42	0.19	2.24	.03^d^	
PTSD diagnosis	0.19	0.06	3.40	.002^e^	
Non-PTSD mental health diagnosis	−0.05	0.05	0.91	.37	
Age (years)	<−0.01	<0.01	0.35	.73	
Probable PTSD (PCL-5^f^≥31) Model					0.21^d^
Intercept	0.43	0.28	1.55	.13	
Probable PTSD (PCL-5≥31)	0.24	0.07	3.30	.003^e^	
Non-PTSD mental health diagnosis	−0.03	0.07	0.44	.66	
Age (years)	<−0.01	<0.01	−0.47	.64	
Probable PTSD (PCL-5≥38) Model					<0.01
Intercept	0.36	0.14	2.53	.02^d^	
Probable PTSD (PCL-5≥38)	<0.01	0.04	0.14	.89	
Non-PTSD mental health diagnosis	0.02	0.04	0.46	.65	
Age (years)	<0.01	<0.01	0.67	.51	

a Linear regression was used to assess the relationship between mean predicted scores from XGBoost models and each outcome (ie, PTSD diagnosis, probable PTSD diagnosis [PCL-5≥31], and probable PTSD diagnosis [PCL-5≥38]), while accounting for age and current non-PTSD mental health diagnosis.

b B values reflect the change in average XGBoost predicted score.

c *R2* values presented are adjusted.

d *P*<.05.

e *P*<.01.

f PCL-5: Posttraumatic Stress Disorder Checklist for *Diagnostic and Statistical Manual of Mental Disorders, Fifth Edition*.

### Probable PTSD Diagnosis (PCL-5≥31)

Three features were selected for the PCL-5≥31 model: efficiency, fragmentation, and restfulness rating (Table S3 in Multimedia Appendix 3). Model performance was satisfactory (Table 3), with an AUC of 0.84 (95% CI 0.71‐0.98), accuracy of 79%, sensitivity of 73% (8/11), specificity of 82% (18/22), precision of 67%, and an *F*_1_-score of 0.70. A bootstrap test indicated that the AUC of the PCL-5≥31 model was not significantly different from that achieved by the PTSD diagnosis model (*P*=.89). Efficiency was the strongest predictor (SHAP=1.38; information gain=0.48), followed by fragmentation (SHAP=1.35; information gain=0.38) and restfulness rating (SHAP=0.92; information gain=0.14).

Linear regression results indicated a significant positive relationship between predicted probability scores and probable PTSD status (B=0.24; *P*.003), with individuals meeting the PCL-5≥31 threshold showing higher predicted scores, around .24 points on average, than those who did not meet this threshold (Table 4). The model accounted for approximately 21% of the variance in predicted scores (adjusted *R²*=0.21). Similar to the diagnosis model, age and non-PTSD diagnoses were not significant predictors (*P* values>.05).

### Probable PTSD Diagnosis (PCL-5≥38)

Three features were selected for the PCL-5≥38 model: restfulness rating, intradaily variability, and fragmentation. As shown in Table 3, this model exhibited the poorest performance, with an AUC of 0.47 (95% CI 0.24‐0.69), accuracy of 61%, sensitivity of 43% (3/7), specificity of 65% (17/26), precision of 25%, and an *F*_1_-score of 0.32. Bootstrap tests demonstrated that the AUC of the PCL-5≥38 model was significantly lower than the AUC of the PCL-5≥31 model (*P*=.004) and the PTSD diagnosis model (*P*=.02). Restfulness rating remained the top predictor (SHAP=0.42; information gain=0.60), followed by intradaily variability (SHAP=0.20; information gain=0.20) and fragmentation (SHAP=0.26; information gain=0.20).

Linear regression revealed no significant association between predicted probability scores and probable PTSD status at the PCL-5≥38 threshold (B<0.01; *P*=.89), with the model explaining a negligible proportion of variance in predicted scores (*R²*<0.01; Table 4). Age and non-PTSD mental health diagnoses were also not significant predictors (*P* values>.05).

## Discussion

This is the first study to explore whether rest, activity, and rest-activity rhythm data, derived from actigraphy and sleep logs, could be used to predict diagnosed PTSD and probable PTSD based on commonly used cutoffs on the PCL-5. The PTSD diagnosis model and the PCL-5≥31 model showed satisfactory performance across model metrics, including accuracy, sensitivity, specificity, and *F*_1_-scores. Bootstrapped tests revealed no significant differences in AUC values across the diagnosis and PCL-5≥31 models. However, performance estimates should be interpreted with caution, due to the nature of the study and the impact of the smaller sample size on estimate precision. Moreover, predicted scores from both models showed a significant positive association with PTSD diagnosis, while accounting for non-PTSD mental health diagnoses. This indicates that average model-predicted probability scores were significantly associated with PTSD diagnosis or probable diagnosis and were not merely predictive of the presence of mental health problems more broadly. However, adjusted *R^2^* values reflect a large portion of unexplained variance in model-predicted probability scores. The findings align with results from a recent meta-analysis examining the performance of tree-based machine learning models in predicting PTSD diagnosis in US military samples (pooled AUC: 0.75; range=0.57-0.92) [41]. Notably, results from these past studies were based on the use of different markers to predict PTSD, namely self-report assessments [42], speech [43], and heart rate data [44]. Prior studies using passively sensed rest-activity and heart rate data to predict probable PTSD (PCL-5≥31) in a national sample of trauma-exposed adults have shown relatively lower model performance (AUCs of 0.64 and 0.56) [21,24]. Greater predictive performance has been achieved in past work using both wrist actigraphy and self-report mental health surveys to predict probable PTSD (AUC=0.73) [21]. In line with this, this exploratory study leveraged both actigraphy data and sleep log data, potentially contributing to improved predictive performance. Notably, the integration of sleep logs may provide a more efficient way to capture important information for predicting PTSD compared to longer self-report batteries. It may be that a combination of both self-report data and wrist actigraphy data can provide greater predictive performance in identifying PTSD through clinical diagnosis and probable PTSD through self-report.

The features that were used as predictors varied across the models, with the top 3 features retained in each to ensure model stability and mitigate overfitting. In the PTSD diagnosis model, subjective restfulness was the most influential predictor. WASO and acrophase time also contributed, suggesting that both sleep quality and circadian rhythm markers are relevant to predicting PTSD diagnosis. In the PCL-5≥31 model, sleep efficiency was the strongest predictor, followed closely by fragmentation, and subjective restfulness. In the PTSD diagnosis model and the PCL-5≥31 model, measures of both subjective and objective features contributed to the overall model prediction. Additionally, among the objective markers, features associated with sleep quality (WASO, sleep efficiency, and fragmentation) were consistently identified as top features. This is consistent with past research, which has demonstrated relationships between PTSD symptoms and the features retained across models in this study, including relationships between PTSD symptoms and markers of sleep fragmentation [18], WASO [19], intradaily variability [18], and acrophase time [45]. However, in this study, feature selection was performed using the entire sample to ensure stability in features. As a result, model performance is likely overly optimistic.

The results of this preliminary study suggest that combining passively sensed sleep data and sleep log data may enhance PTSD symptom assessment and monitoring. In line with this, past research has demonstrated different relationships between PTSD symptoms and objective and subjective measures of rest-activity rhythms [13,14,16,18,19]. Findings from this study may support the idea that subjective and objective rest-activity markers capture distinct phenomena and differ in their use for predicting PTSD symptoms. Furthermore, the results may suggest that both subjective and objective data can offer greater benefits than relying on either in isolation. For example, combining subjective and objective data may help identify markers of paradoxical insomnia, which is often observed in individuals with clinically significant PTSD symptoms [46]. However, given the sample size and scope of this study, further research is needed to validate these findings beyond this small study.

The model predicting probable PTSD at the more conservative cutoff (PCL-5≥38) demonstrated relatively poor performance in this sample. Bootstrap test results reflected that the AUC value for the PCL-5≥38 model was significantly lower than the AUC value achieved by the diagnosis and the PCL-5≥31 models. Importantly, fewer individuals met the more conservative PCL-5 cutoff in this study (n=7; approximately 35 days of data), resulting in potentially greater difficulty in distinguishing individuals based on this outcome, compared to the more liberal cutoff (n=11). Interestingly, this model shared overlapping features with the other 2 models, including restfulness and fragmentation. However, SHAP values were much lower for these features, indicating that they were less meaningful in the overall prediction of a more conservative PCL-5 cutoff. Unlike the other 2 models, the PCL-5≥38 model also included intradaily variability, which captures variability in rest-activity rhythms (ie, how often transitions occur between rest and activity within a day). Potentially, those reporting more severe symptoms, but not meeting criteria for a clinical diagnosis, may have more erratic rest-activity patterns that make predictions from rest-activity metrics more difficult. Future work should consider using longitudinal data to capture the relationship between symptom trajectories and rest-activity rhythm data over time rather than relying on predicting symptoms at a single time point. In particular, research may benefit from more dynamic prediction methods (eg, deep learning using rest-activity data) to effectively predict PTSD symptoms. By capturing these data, it may become evident that the predictive use of different markers varies for individuals across time. For example, the predictive use of subjective restfulness may be greater for individuals who experience more persistent circadian rhythm disruption across time.

There are several important limitations to consider when interpreting these findings. First, the sample size was relatively small and consisted exclusively of male-identifying service members and veterans who had experienced trauma in the context of military service. In contrast, prior studies have developed predictive models using larger and more heterogeneous samples [21]. Only 2 cutoffs (PCL-5≥31 and ≥38) were used in this study; however, a range of potential cutoffs has been identified [29]. This limits the understanding of the predictive performance of these models across different potential outcomes, which may also vary based on context, population, and goal. Notably, rest-activity disturbances are evaluated through both self-report and clinician-administered assessments for PTSD. As this study used the subjective and objective rest-activity data to predict PTSD-specific outcomes, there may be an elevated risk of circularity in the relationships between predictors and each outcome.

This study used a 2-stage modeling approach in which feature selection was conducted on aggregated person-level data across the full sample prior to model training and testing using a leave-one-subject-out cross-validation procedure on day-level data. This approach was chosen to ensure the stability of feature selection in light of the small sample size. However, this results in data leakage, resulting in upwardly-biased model performance estimates. While univariate feature selection was used to reduce the risk of overfitting, these results provide less understanding of multivariate interactions. Future research with larger and more diverse samples is necessary to validate these findings and further explore complex interactions between features. Model performance may be biased due to the sample size and feature selection methods. In addition, validating model performance in independent clinical samples is specifically needed. Including other objective rest-activity metrics (eg, mean activity during the 5 least active hours of the day and mean activity during the 10 most active hours of the day) may enhance model performance and provide a more comprehensive understanding of the relationship between rest-activity patterns and PTSD symptoms. Furthermore, future research may benefit from using individualized modeling approaches to assess the use of intraindividual prediction and the relative importance of features on an individual basis. This may offer more clinically relevant insights into how specific patterns of circadian rhythm disruption relate to PTSD symptoms at the individual level, which in turn can create individualized forms of assessment, monitoring, and intervention. For example, this may support the implementation of tailored chronotherapy (eg, just-in-time interventions focused on regulating one’s circadian rhythm and time-based reminders for certain medications based on individual rest-activity rhythms) to improve clinical outcomes for individuals with PTSD symptoms. Moreover, actigraphy data used in this study captured only a single week of time. Some evidence suggests that differing periods of monitoring may significantly improve the reliability and predictive power of rest-activity models for other mental health concerns (eg, depression) [22]. PTSD symptoms are dynamic across time, and thus, greater periods of time may be needed to capture symptom trends [27,28]. As such, future work would benefit from investigating the optimal window of time needed to capture rest-activity patterns most predictive of PTSD symptoms. This is particularly important for developing scalable and clinically meaningful forms of assessment, monitoring, and intervention that integrate objective and subjective rest-activity data.

Ultimately, the machine learning classifier used in this study demonstrated satisfactory performance in predicting both clinician-diagnosed PTSD and probable PTSD using the more liberal self-report threshold (PCL-5≥31) in this small sample, leveraging a combination of subjective and objective rest-activity data. In contrast, the model predicting probable PTSD based on the more conservative cutoff (PCL-5≥38) demonstrated poor performance. These results highlight differences in the rest-activity markers most relevant to clinically-diagnosed PTSD versus probable PTSD based on self-report. Future research should aim to examine the integration of wearable technology and real-time naturalistic assessment to evaluate dynamic methods of PTSD assessment involving rest-activity rhythm data. Ultimately, integrating both subjective and objective measures of rest-activity may offer a more comprehensive and accurate approach to predicting PTSD.

## Supplementary material

10.2196/86025 Multimedia Appendix 1 Wrist actigraphy features.

10.2196/86025 Multimedia Appendix 2 Correlations between rest-activity features.

10.2196/86025 Multimedia Appendix 3 *F*_1_-scores of aggregated features with each outcome.

10.2196/86025 Multimedia Appendix 4 Extreme gradient boosting (XGBoost) model hyperparameters.

10.2196/86025 Multimedia Appendix 5 Logistic regression results.

## Abbreviations

AUC: area under the curve
DSM-5: *Diagnostic and Statistical Manual of Mental Disorders, Fifth Edition*
PCL-5: Posttraumatic Stress Disorder Checklist for Diagnostic and Statistical Manual of Mental Disorders, Fifth Edition
PTSD: posttraumatic stress disorder
SCID-5-RV: Structured Clinical Interview for DSM-5 Disorders–Research Version
SHAP: Shapley Additive Explanations
WASO: wake after sleep onset
XGBoost: extreme gradient boosting
