# Supplementary material for: Detection of Posttraumatic Stress Disorder With Rest-Activity Data: Machine Learning Approach Using Wearable and Self-Report Data
Source: JMIR Form Res. 2026 May 19;10:e86025. doi: 10.2196/86025 (PMC13186518; doi:10.2196/86025)
Supplement: Multimedia Appendix 1 [file formative-v10-e86025-s001.docx]

**Supplementary Table 1.** *Wrist actigraphy features.*

| **Feature** | **Definition** |
| --- | --- |
| **Mesor** | The mean level of the fitted 24-hour activity rhythm, representing the baseline activity level. |
| **Acrophase Time** | The decimal time value corresponding to the peak of the 24-hour activity rhythm. |
| **Amplitude** | The difference between the peak and the mesor of the fitted 24-hour rhythm, reflecting the strength of the circadian rhythm. |
| **Relative Amplitude** | The normalized difference between the most and least active periods. |
| **Mean Activity** | The average activity level across the recording period. |
| **Standard Deviation of Activity** | The variability in activity levels across the recording period. |
| **Root Mean Square Successive Difference (RMSSD)** | A measure of short-term variability in activity, calculated as the square root of the mean squared differences between successive activity readings. |
| **Intradaily Variability** | A measure of fragmentation in the rest-activity rhythm, indicating how often transitions occur between rest and activity within a day. |
| **Interdaily Stability** | A measure of how consistent the 24-hour activity pattern is across multiple days, with higher values indicating stronger synchronization to the activity cycle. |
| **M10** | The mean activity level during the 10 most active consecutive hours of the day. |
| **L5** | The mean activity level during the 5 least active consecutive hours of the day. |
| **Circadian Rhythm Strength** | A general metric quantifying the overall regularity and amplitude of the circadian activity rhythm (M10/L5). |
| **Total Sleep Time (TST)** | The total amount of time in minutes scored as sleep during the main sleep period. |
| **Efficiency** | Proportion of time between identified sleep onset and final wake time scored as sleep, multiplied by 100. |
| **Wake After Sleep Onset (WASO)** | The total amount of time spent awake after initially falling asleep, indicating sleep fragmentation. |
| **Fragmentation** | The sum of percent mobile and percent one minute immobile bouts divided by the number of immobile bouts for the given interval. |
