# Supplementary material for: Detection of Posttraumatic Stress Disorder With Rest-Activity Data: Machine Learning Approach Using Wearable and Self-Report Data
Source: JMIR Form Res. 2026 May 19;10:e86025. doi: 10.2196/86025 (PMC13186518; doi:10.2196/86025)
Supplement: Multimedia Appendix 2 [file formative-v10-e86025-s002.docx]

**Supplementary Table 2.** *Correlations between rest-activity features.*

| **Variable** | **1** | **2** | **3** | **4** | **5** | **6** | **7** | **8** | **9** | **10** | **11** | **12** | **13** | **14** | **15** | **16** | **17** |
| --- | --- | --- | --- | --- | --- | --- | --- | --- | --- | --- | --- | --- | --- | --- | --- | --- | --- |
| 1. Mesor |  |  |  |  |  |  |  |  |  |  |  |  |  |  |  |  |  |
| 2. Acrophase Time | -.11 |  |  |  |  |  |  |  |  |  |  |  |  |  |  |  |  |
| 3. Amplitude | .85*** | .03 |  |  |  |  |  |  |  |  |  |  |  |  |  |  |  |
| 4. Relative Amplitude | .14 | .08 | .35* |  |  |  |  |  |  |  |  |  |  |  |  |  |  |
| 5. Mean Activity | .84*** | -.14 | .65*** | .20 |  |  |  |  |  |  |  |  |  |  |  |  |  |
| 6. SD Activity | .79*** | -.17 | .69*** | .25 | .94*** |  |  |  |  |  |  |  |  |  |  |  |  |
| 7. CRS | .12 | .03 | .25 | .80*** | .19 | .21 |  |  |  |  |  |  |  |  |  |  |  |
| 8. M10 | .86*** | .03 | .82*** | .35* | .94*** | .92*** | .32 |  |  |  |  |  |  |  |  |  |  |
| 9. L5 | .29 | -.10 | .04 | -.82*** | .28 | .22 | -.70*** | .14 |  |  |  |  |  |  |  |  |  |
| 10. RMSSD | .76*** | -.26 | .56*** | .12 | .93*** | .95*** | .11 | .83*** | .31 |  |  |  |  |  |  |  |  |
| 11. TST | -.51** | .07 | -.15 | .55*** | -.41* | -.25 | .35* | -.26 | -.75*** | -.36* |  |  |  |  |  |  |  |
| 12. IV | -.36* | -.04 | -.62*** | -.56*** | -.35* | -.37* | -.42* | -.50** | .29 | -.27 | -.24 |  |  |  |  |  |  |
| 13. IS | .35* | -.05 | .53** | .62*** | .23 | .25 | .35* | .34 | -.40* | .19 | .30 | -.64*** |  |  |  |  |  |
| 14. Sleep Quality Rating | -.06 | -.15 | .10 | .34 | -.14 | -.09 | .20 | -.05 | -.31 | -.11 | .32 | -.14 | .36* |  |  |  |  |
| 15. Restfulness Rating | -.17 | -.05 | -.01 | .26 | -.17 | -.10 | .15 | -.09 | -.21 | -.11 | .34 | -.09 | .29 | .91*** |  |  |  |
| 16. WASO | -.36* | .05 | -.28 | -.42* | -.28 | -.23 | -.48** | -.31 | .18 | -.21 | .31 | .20 | -.24 | -.32 | -.17 |  |  |
| 17. Efficiency | -.16 | -.04 | .10 | .59*** | -.23 | -.11 | .45** | -.08 | -.60*** | -.19 | .51** | -.27 | .25 | .49** | .37* | -.46** |  |
| 18. Fragmentation | -.32 | .13 | -.40* | -.58*** | -.27 | -.38* | -.44* | -.34 | .30 | -.31 | -.02 | .45** | -.44** | -.27 | -.15 | .74*** | -.63*** |

Note. SD = Standard Deviation; CRS = Circadian Rhythm Strength; M10 = Mean of ten most active hours of the day; L5 = mean of the five least active hours of the day; RMSSD = Root mean square of successive differences; TST = Total Sleep Time; IV = Intradaily Variability; IS = Interdaily Stability; WASO = Wake after sleep onset.

**p* < .05, ***p* < .01, ****p* < .001.
