# Supplementary material for: Detection of Posttraumatic Stress Disorder With Rest-Activity Data: Machine Learning Approach Using Wearable and Self-Report Data
Source: JMIR Form Res. 2026 May 19;10:e86025. doi: 10.2196/86025 (PMC13186518; doi:10.2196/86025)
Supplement: Multimedia Appendix 3 [file formative-v10-e86025-s003.docx]

**Supplementary Table 3.** *F-scores of aggregated features with each outcome.*

| **Feature** | **PTSD Diagnosis** | **PCL-5 ≥ 31** | **PCL-5 ≥ 38** |
| --- | --- | --- | --- |
| **Sleep Quality Rating** | 4.49, p = .043 | 3.38, p = .076 | 1.21, p = .279 |
| **Rest Rating** | **5.67, p = .024** | **6.78, p = .014** | **4.20, p = .049** |
| **Acrophase Time** | **3.55, p = .069** | 1.82, p = .187 | 0.37, p = .546 |
| **WASO** | **2.28, p = .141** | 0.80, p = .378 | 1.09, p = .304 |
| **L5** | 1.23, p = .276 | 0.50, p = .484 | 0.03, p = .856 |
| **Fragmentation** | 1.33, p = .258 | **2.64, p = .114** | **2.15, p = .153** |
| **M10** | 0.28, p = .598 | 1.15, p = .293 | 0.94, p = .340 |
| **CRS** | 0.44, p = .510 | 0.13, p = .725 | 1.19, p = .285 |
| **TST** | 0.61, p = .442 | 1.23, p = .276 | 0.00, p = .982 |
| **Mean Activity** | 0.13, p = .724 | 2.22, p = .147 | 0.55, p = .464 |
| **IV** | 0.14, p = .713 | 0.40, p = .533 | **4.49, p = .042** |
| **SD Activity** | 0.05, p = .833 | 0.94, p = .340 | 0.38, p = .543 |
| **Mesor** | 0.05, p = .833 | 1.08, p = .308 | 0.91, p = .349 |
| **Efficiency** | 0.05, p = .821 | **4.23, p = .048** | 0.12, p = .735 |
| **RMSSD** | 0.03, p = .873 | 0.99, p = .326 | 0.35, p = .558 |
| **Relative Amplitude** | 0.14, p = .707 | 0.87, p = .357 | 1.89, p = .179 |
| **Amplitude** | 0.00, p = .945 | 0.20, p = .659 | 1.61, p = .214 |

Note. Univariate relationships between aggregated features and each outcome. SD = Standard Deviation; CRS = Circadian Rhythm Strength; M10 = Mean of ten most active hours of the day; L5 = mean of the five least active hours of the day; RMSSD = Root mean square of successive differences; TST = Total Sleep Time; IV = Intradaily Variability; WASO = Wake after sleep onset. Bolded cells reflect the selected features for the subsequent XGBoost models.
