# Supplementary material for: Detection of Posttraumatic Stress Disorder With Rest-Activity Data: Machine Learning Approach Using Wearable and Self-Report Data
Source: JMIR Form Res. 2026 May 19;10:e86025. doi: 10.2196/86025 (PMC13186518; doi:10.2196/86025)
Supplement: Multimedia Appendix 4 [file formative-v10-e86025-s004.docx]

**Supplementary Table 4.** *XGBoost model hyperparameters.*

|  | **PTSD Diagnosis Model** | **PCL-5 ≥ 31 Model** | **PCL-5 ≥ 38**  **Model** |
| --- | --- | --- | --- |
| **Eta** | 0.049622475 | 0.06087177 | 0.01130688 |
| **Maximum Depth** | 2 | 4 | 2 |
| **Minimum Child Weight** | 4 | 2 | 4 |
| **Rounds** | 100 | 590 | 200 |
| **Max Delta Step** | 1 | 1 | 1 |

Note. PTSD = Posttraumatic Stress Disorder; PCL-5 = Posttraumatic Checklist for DSM-5. Hyperparameters were optimized using random grid search. Eta (learning rate) controls the contribution of each individual tree to the final prediction. Maximum depth determines the depth for each individual decision tree. Minimum child weight sets the minimum sum of instance weights per each child node. Rounds refer to the number of boosting iterations. Max delta step restricts the maximum change allowed in the weight estimation at each boosting step.
