# Supplementary material for: Detection of Posttraumatic Stress Disorder With Rest-Activity Data: Machine Learning Approach Using Wearable and Self-Report Data
Source: JMIR Form Res. 2026 May 19;10:e86025. doi: 10.2196/86025 (PMC13186518; doi:10.2196/86025)
Supplement: Multimedia Appendix 5 [file formative-v10-e86025-s005.docx]

**Supplemental Table 5.** *Logistic regression results*

| **Variable** | **OR** | **SE** | ***z*** | ***p*** | ***Nagelkerke R²*** |
| --- | --- | --- | --- | --- | --- |
| **PTSD Diagnosis** |  |  |  |  | **.42** |
| Intercept | -7.65 | 0.19 | -1.17 | .24 |  |
| WASO | 0.19 | 0.03 | 0.59 | .55 |  |
| Restfulness Rating | -1.11 | 0.57 | -1.93 | .05 |  |
| Acrophase Time | 0.79 | 0.47 | 1.67 | .09 |  |
| **Probable PTSD (PCL-5 ≥ 31)** |  |  |  |  | **.33** |
| Intercept | 5.89 | 8.92 | 0.66 | .51 |  |
| Fragmentation | 0.05 | 0.08 | 0.67 | .50 |  |
| Efficiency | -0.04 | 0.10 | 0.37 | .71 |  |
| Restfulness Rating | -0.82 | 0.44 | 1.89 | .06 |  |
| **Probable PTSD (PCL-5 ≥ 38)** |  |  |  |  | **.39** |
| Intercept | 0.11 | 3.44 | 0.03 | .97 |  |
| IV | 5.42 | 3.53 | 1.54 | .12 |  |
| Fragmentation | -0.01 | 0.08 | 0.12 | .90 |  |
| Restfulness Rating | 1.20 | 0.63 | 1.89 | .06 |  |

Note. Logistic regression was used to assess the relationship between features and outcomes. OR = odds ratio; PTSD = Posttraumatic Stress Disorder; WASO = Wake after sleep onset; PCL-5 = Posttraumatic Checklist for DSM-5; IV = Intradaily Variability.

**p* < .05, ***p* < .01, ****p* < .001.
